# Pleuritis

**Published:** 1842-07-02

**Authors:** 


*Pleuritis.*—Of this four cases were treated,—one white and three blacks. Two of these cases were uncomplicated; they passed through their various stages rapidly, and convalesced in about ten days. The other cases were complicated, and terminated fatally.

The first case, J. M., ætat. 30, an Irishman, of middle stature, and embonpoint, entered the ward with *pericarditis* and pleuritis of both sides. At the time of his entrance (two weeks after his date of attack,) the effusion was slight, but it rapidly increased, producing extreme dyspnœa, flushed face, etc. The effusion, as measured by the physical signs, occupied the whole of the lower half of the chest. At the upper part of the liquid, œgophony was distinctly heard. The sounds of the heart were entirely lost at the apex.

He was bled from the arm when he first entered, and given blue mass, with Dover's powder, till slight ptyalism was induced. When the inflammatory symptoms passed off, blisters were applied to his chest, and kept constantly discharging. This plan of treatment was continued for about four weeks, and with very great improvement. The effusion was diminished so that he could recline on either side without inconvenience. His appetite was tolerable, and his skin moist and of natural temperature, but his pulse still ranged from 100 to 120, and his respiratian from 28 to 36 a minute. He now (four weeks after his entrance in the ward,) became suddenly delirious; his mind wandered; he had great difficulty in finishing words; his pupils were dilated; the expression frowning; and the features distorted; and also great stupor, with sleepiness. After these symptoms appeared, he was cupped and blisters applied to the nucha, a bladder of ice was applied to his head, and a warm stimulating pediluvium given night and morning. At the end of a few weeks he convalesced from the cerebral affection, and erysipelas appeared involving the upper part of the face and most of the scalp. This readily yielded to gentle laxatives, local applications of flaxseed, mucilage, and good diet, with about four ounces of wine a day. But at the conclusion of this last affection, the cerebral symptoms were again excited and gradually increased until he died, which was about two months after his first entrance in the hospital.

Thursday previous to death, the effusion in the chest increased very considerably, and continued increasing till he died.

On examination of the body about twelve hours after death, the arachnoid membrane was found opaque, with stellated spots of bright red injection over both hemispheres of the cerebrum on the upper part. At the base of the brain the same condition was found but in a less degree, The substance of the brain was natural, and no effusion in the ventricles. The cerebellum was natural.

Each *pleura* contained about three quarts of yellowish serum. The right pleura was converted into a hard leathery membrane, covered with a deposit of soft lymph. There was also an adventitious membrane between the pleuræ. The right lung was about one-fourth the usual size. Internally it was intensely red and marbled in appearance, with semi-transparent granules scattered throughout.

The left pleura was thickened like the right, with more lymph effused on it, and much better organised. The lung was very much congested, and had a few scattered tubercles in it.

The *pericardium* contained a considerable amount of yellowish serum, perhaps a pint.

[*Remarks.*—This case did not terminate fatally from the pleurisy, notwithstanding it was double and extremely serious. There was, on the contrary, at one period, a reasonable prospect of recovery, but the constitution of the patient was broken down, partly from causes which preceded his disease, partly from its extent, and finally, from the arachnitis and erysipelas of the face.

There was one point of some pathological interest. In the majority of cases double pleurisy is complicated with tubercles in the lung, but in the present case, there was every reason to believe that the lungs did not become tuberculous until long after the pleuritic effusion had formed.

The usual treatment for pleurisy is apt to fail when the disease is double, not only from the severity of the affection, but because the affected lung is kept in continual movement, while in those cases in which the inflammation is confined to one side, the partial cessation of respiration on the inflamed side certainly contributes greatly to the cure of the disease, and the consolidation of the lymph.—W. W. G.]

